# Supplementary material for: Discovery and application of insertion-deletion (INDEL) polymorphisms for QTL mapping of early life-history traits in Atlantic salmon
Source: BMC Genomics. 2010 Mar 8;11:156. doi: 10.1186/1471-2164-11-156 (PMC2838853; doi:10.1186/1471-2164-11-156)
Supplement: Additional file 2 — Information on developed 76 locus single-run INDEL panel in Atlantic salmon. Information on fluorescence labeling, primer concentrations, PCR pooling and links to alignments, INDEL motifs and GENESCAN (Burge and Karlin 1997) predictions of genes/exons are available in html format. [file 1471-2164-11-156-S2.ZIP › Additionalfile2/table17300.html]

Contig Alignment

```
Base	A B C D E F   
1	. A A A A A    
2	. G G G G G    
3	. T T T T T    
4	. A A A A A    
5	. T T T T T    
6	. G G G G G    
7	. A A A A A    
8	. T T T T T    
9	. A A A A A    
10	. T T T T T    
11	. G G G G G    
12	. T T T T T    
13	. T T T T T    
14	. A A A A A    
15	. T T T T T    
16	. G G G G G    
17	. T T T T T    
18	. T T T T T    
19	. T T T T T    
20	. A A A A A    
21	. A A A A A    
22	. T T T T T    
23	. A A A A A    
24	. T T T T T    
25	. G G G G G    
26	. G G G G G    
27	. T T T T T    
28	. T T T T T    
29	. A A A A A    
30	. C C C C C    
31	. A A A A A    
32	. T T T T T    
33	. A A A A A    
34	. A A A A A    
35	. G G G G G    
36	A A A A A A    
37	C C C C C C    
38	A A A A A A    
39	G G G G G G    
40	A A A A A A    
41	A A A A A A    
42	C C C C C C    
43	A A A A A A    
44	C C C C C C    
45	A A A A A A    
46	G G G G G G    
47	C C C C C C    
48	T T T T T T    
49	A A A A A A    
50	A A A A A A    
51	C C C C C C    
52	T T T T T T    
53	T T T T T T    
54	T T T T T T    
55	A A A A A A    
56	G G G G G G    
57	C C C C C C    
58	A A A A A A    
59	T T T T T T    
60	T T T T T T    
61	T T T T T T    
62	C C C C C C    
63	A A A A A A    
64	G G G G G G    
65	C C C C C C    
66	T T T T T T    
67	A A A A A A    
68	T T T T T T    
69	T T T T T T    
70	A A A A A A    
71	A A A A A A    
72	A A A A A A    
73	C C C C C C    
74	A A A A A A    
75	A A A A A A    
76	C C C C C C    
77	T T T T T T    
78	T T T T T T    
79	T T T T T T    
80	T T T T T T    
81	C C C C C C    
82	A A A A A A    
83	A A A A A A    
84	C C C C C C    
85	T T T T T T    
86	A A A A A A    
87	C C C C C C    
88	T T T T T T    
89	T T T T T T    
90	A A A A A A    
91	C C C C C C    
92	T T T T T T    
93	A A A A A A    
94	C C C C C C    
95	T T T T T T    
96	T T T T T T    
97	T T T T T T    
98	T T T T T T    
99	T T T T T T    
100	A A A A A A    
101	G G G G G G    
102	C C C C C C    
103	T T T T T T    
104	A A A A A A    
105	C C C C C C    
106	T T T T T T    
107	T T T T T T    
108	T T T T T T    
109	G G G G G G    
110	C C C C C C    
111	A A A A A A    
112	A A A A A A    
113	C C C C C C    
114	T T T T T T    
115	A A A A A A    
116	C C C C C C    
117	A A A A A A    
118	T T T T T T    
119	A A A A A A    
120	G G G G G G    
121	C C C C C C    
122	A A A A A A    
123	T T T T T T    
124	G G G G G G    
125	T T T T T T    
126	T T T T T T    
127	A A A A A A    
128	G G G G G G    
129	C C C C C C    
130	T T T T T T    
131	A A A A A A    
132	A A A A A A    
133	C C C C C C    
134	C C C C C C    
135	C C C C C C    
136	T T T T T T    
137	T T T T T T    
138	C C C C C C    
139	C C C C C C    
140	C C C C C C    
141	C C C C C C    
142	T T T T T T    
143	A A A A A A    
144	T T T T T T    
145	C C C C C C    
146	C C C C C C    
147	T T T T T T    
148	T T T T T T    
149	A A A A A A    
150	A A A A A A    
151	C C C C C C    
152	C C C C C C    
153	C C C C C C    
154	T T T T T T    
155	A A A A A A    
156	A A A A A A    
157	A A A A A A    
158	C C C C C C    
159	G G G G G G    
160	T T T T T T    
161	A A A A A A    
162	A A A A A A    
163	G G G G G G    
164	C C C C C C    
165	C C C C C C    
166	T T T T T T    
167	T T T T T T    
168	T T T T T T    
169	T T T T T T    
170	A A A A A A    
171	G G G G G G    
172	C C C C C C    
173	T T T T T T    
174	A A A A A A    
175	A A A A A A    
176	C C C C C C    
177	C C C C C C    
178	T T T T T T    
179	T T T T T T    
180	C C C C C C    
181	C C C C C C    
182	C C C C C C    
183	C C C C C C    
184	T T T T T T    
185	A A A A A A    
186	A A A A A A    
187	C C C C C C    
188	C C C C C C    
189	C C C C C C    
190	T T T T T T    
191	G G G G G G    
192	T T T T T T    
193	A A A A A A    
194	A A A A A A    
195	C C C C C C    
196	C C C C C C    
197	T T T T T T    
198	T T T T T T    
199	A A A A A A    
200	A A A A A A    
201	C C C C C C    
202	C C C C C C    
203	C C C C C C    
204	T T T T T T    
205	A A A A A A    
206	A A A A A A    
207	C C C C C C    
208	C C C C C C    
209	C C C C C C    
210	C C C C C C    
211	T T T T T T    
212	A A A A A A    
213	T T T T T T    
214	C C C C C C    
215	C C C C C C    
216	T T T T T T    
217	T T T T T T    
218	A A A A A A    
219	A A A A A A    
220	C C C C C C    
221	T T T T T T    
222	C C C C C C    
223	T T T T T T    
224	T T T T T T    
225	A A A A A A    
226	G G G G G G    
227	C C C C C C    
228	C C C C C C    
229	T T T T T T    
230	A A A A A A    
231	A A A A A A    
232	C C C C C C    
233	T T T T T T    
234	A A A A A A    
235	A A A A A A    
236	- - - - C C  2	 indel  
237	- - - - T T  2	 indel  
238	- - - - A A  2	 indel  
239	- - - - A A  2	 indel  
240	C C C C C C    
241	G G G G G G    
242	C C C C C C    
243	T T T T T T    
244	A A A A A A    
245	G G G G G G    
246	C C C C C C    
247	C C C C C C    
248	A A A A A A    
249	G G G G G G    
250	C C C C C C    
251	T T T T T T    
252	A A A A A A    
253	G G G G G G    
254	C C C C C C    
255	T T T T T T    
256	A A A A A A    
257	A A A A A A    
258	T T T T T T    
259	G G G G G G    
260	T T T T T T    
261	T T T T T T    
262	A A A A A A    
263	A A A A A A    
264	C C C C C C    
265	G G G G G G    
266	T T T T T T    
267	T T T T T T    
268	A A A A A A    
269	G G G G G G    
270	C C C C C C    
271	C C C C C C    
272	A A A A A A    
273	C C C C C C    
274	C C C C C C    
275	T T T T T T    
276	A A A A A A    
277	G G G G G G    
278	C C C C C C    
279	T T T T T T    
280	C C C C C C    
281	A A A A A A    
282	C C C C C C    
283	A A A A A A    
284	T T T T T T    
285	T T T T T T    
286	A A A A A A    
287	G G G G G G    
288	C C C C C C    
289	A A A A A A    
290	A A A A A A    
291	C C C C C C    
292	A A A A A A    
293	T T T T T T    
294	G G G G G G    
295	A A A A A A    
296	A A A A A A    
297	A A A A A A    
298	T T T T T T    
299	T T T T T T    
300	G G G G G G    
301	G G G G G G    
302	A A A A A A    
303	A A A A A A    
304	T T T T T T    
305	T T T T T T    
306	T T T T T T    
307	G G G G G G    
308	T T T T T T    
309	A A A A A A    
310	A A A A A A    
311	C C C C C C    
312	A A A A A A    
313	T T T T T T    
314	A A A A A A    
315	T T T T T T    
316	C C C C C C    
317	C C C C C C    
318	T T T T T T    
319	A A A A A A    
320	A A A A A A    
321	A A A A A A    
322	T T T T T T    
323	A A A A A A    
324	T T T T T T    
325	T T T T T T    
326	T T T T T T    
327	C C C C C C    
328	A A A A A A    
329	A A A A A A    
330	A A A A A A    
331	T T T T T T    
332	T T T T T T    
333	C C C C C C    
334	G G G G G G    
335	T T T T T T    
336	A A A A A A    
337	G G G G G G    
338	C C C C C C    
339	A A A A A A    
340	T T T T T T    
341	A A A A A A    
342	T T T T T T    
343	T T T T T T    
344	C C C C C C    
345	T T T T T T    
346	A A A A A A    
347	A A A A A A    
348	C C C C C C    
349	A A A A A A    
350	C C C C C C    
351	A A A A A A    
352	T T T T T T    
353	T T T T T T    
354	A A A A A A    
355	A A A A A A    
356	T T T T T T    
357	A A A A A A    
358	C C C C C C    
359	A A A A A A    
360	T T T T T T    
361	A A A A A A    
362	C C C C C C    
363	C C C C C C    
364	A A A A A A    
365	T T T T T T    
366	A A A A A A    
367	C C C C C C    
368	A A A A A A    
369	A A A A A A    
370	A A A A A A    
371	A A A A A A    
372	T T T T T T    
373	G G G G G G    
374	T T T T T T    
375	A A A A A A    
376	A A A A A A    
377	C C C C C C    
378	A A A A A A    
379	T T T T T T    
380	C C C C C C    
381	C A A A A A    
382	C C C C C C    
383	A A A A A A    
384	C C C C C C    
385	T T T T T T    
386	A A A A A A    
387	A A A A A A    
388	A A A A A A    
389	T T T T T T    
390	G G G G G G    
391	G G G G G G    
392	A A A A A A    
393	A A A A A A    
394	T T T T T T    
395	G G G G G G    
396	T T T T T T    
397	C C C C C C    
398	T T T T T T    
399	T T T T T T    
400	G G G G G G    
401	G G G G G G    
402	A A A A A A    
403	T T T T T T    
404	A A A A A A    
405	T T T T T T    
406	A A A A A A    
407	C C C C C C    
408	A A A A A A    
409	G G G G G G    
410	T T T T T T    
411	A A A A A A    
412	C C C C C C    
413	A A A A A A    
414	T T T T T T    
415	A A A A A A    
416	C C C C C C    
417	A A A A A A    
418	G G G G G G    
419	A A A A A A    
420	A A A A A A    
421	T T T T T T    
422	A A A A A A    
423	A A A A A A    
424	T T T T T T    
425	A A A A A A    
426	T T T T T T    
427	G G G G G G    
428	A A A A A A    
429	A A A A A A    
430	A A A A A A    
431	T T T T T T    
432	G G G G G G    
433	T T T T T T    
434	T T T T T T    
435	C C C C C C    
436	T T T T T T    
437	T T T T T T    
438	A A A A A A    
439	G G G G G G    
440	A A A A A A    
441	C C C C C C    
442	C C C C C C    
443	A A A A A A    
444	G G G G G G    
445	G G G G G G    
446	T T T T T T    
447	T T T T T T    
448	G G G G G G    
449	G G G G G G    
450	T T T T T T    
451	G G G G G G    
452	A A A A A A    
453	T T T T T T    
454	T T T T T T    
455	A A A A A A    
456	T T T T T T    
457	T T T T T T    
458	T T T T T T    
459	T T T T T T    
460	C C C C C C    
461	T T T T T T    
462	G G G G G G    
463	A A A A A A    
464	C C C C C C    
465	C C C C C C    
466	T T T T T T    
467	G G G G G G    
468	G G G G G G    
469	T T T T T T    
470	A A A A A A    
471	G G G G G G    
472	T T T T T T    
473	T T T T T T    
474	G G G G G G    
475	A A A A A A    
476	C C C C C C    
477	T T T T T T    
478	C C C C C C    
479	T T T T T T    
480	T T T T T T    
481	T T T T T T    
482	C C C C C C    
483	T C T T T T    
484	G G G G G G    
485	A A A A A A    
486	G G G G G G    
487	C C C C C C    
488	T T T T T T    
489	G G G G G G    
490	G G G G G G    
491	G G G G G G    
492	A A A A A A    
493	G G G G G G    
494	T T T T T T    
495	T T T T T T    
496	T T T T T T    
497	A A A A A A    
498	C C C C C C    
499	T T T T T T    
500	C C C C C C    
501	T T T T T T    
502	T T T T T T    
503	T T T T T T    
504	C C C C C C    
505	T T T T T T    
506	G G G G G G    
507	A A A A A A    
508	T T T T T T    
509	T T T T T T    
510	T T T T T T    
511	G G G G G G    
512	G G G G G G    
513	G G G G G G    
514	A A A A A A    
515	G G G G G G    
516	T T T T T T    
517	T T T T T T    
518	G G G G G G    
519	A A A A A A    
520	C C C C C C    
521	T T T T T T    
522	C C C C C C    
523	T T T T T T    
524	T T T T T T    
525	T T T T T T    
526	C C C C C C    
527	C T T T T T    
528	. G G G G G    
529	. A A A A A    
530	. G G G G G    
531	. C C C C C    
532	. T T T T T    
533	. G G G G G    
534	. G G G G G    
535	. A A A A A    
536	. A A A A A    
537	. G G G G G    
538	. T T T T T    
539	. T T T T T    
540	. G G G G G    
541	. A A A A A    
542	. C C C C C    
543	. C C C C C    
544	. C C C C C    
545	. T T T T T    
546	. T T T T T    
547	. T T T T T    
548	. G G G G G    
549	. T T T T T    
550	. G G G G G    
551	. A A A A A    
552	. C C C C C    
553	. C C C C C    
554	. T T T T T    
555	. G G G G G    
556	. G G G G G    
557	. G G G G G    
558	. A A A A A    
559	. G G G G G    
560	. T T T T T    
561	. T T T T T    
562	. G G G G G    
563	. A A A A A    
564	. C C C C C    
565	. T T T T T    
566	. C C C C C    
567	. T T T T T    
568	. T T T T T    
569	. C C C C C    
570	. C C C C C    
571	. T T T T T    
572	. G G G G G    
573	. A A A A A    
574	. C C C C C    
575	. C C C C C    
576	. T T T T T    
577	. . G G G G    
578	. . G G G G    
579	. . T T T T    
580	. . A A A A    
581	. . G G G G    
582	. . T T C C  2	 transition  
583	. . T T T T    
584	. . G G G G    
585	. . A A A A    
586	. . C C C C    
587	. . C C C C    
588	. . T T T T    
589	. . T T T T    
590	. . T T T T    
591	. . T T T T    
592	. . C C C C    
593	. . T T T T    
594	. . G G G G    
595	. . A A A A    
596	. . C C C C    
597	. . C C C C    
598	. . T T T T    
599	. . G G G G    
600	. . G G G G    
601	. . G G G G    
602	. . A A A A    
603	. . G G G G    
604	. . T T T T    
605	. . T T T T    
606	. . G G G G    
607	. . A A A A    
608	. . T T T T    
609	. . T T T T    
610	. . C C C C    
611	. . A A A A    
612	. . T T T T    
613	. . T T T T    
614	. . G G G G    
615	. . A A A A    
616	. . C C C C    
617	. . C C C C    
618	. . T T T T    
619	. . G G G G    
620	. . G G G G    
621	. . A A A A    
622	. . A A A A    
623	. . C C C C    
624	. . T T T T    
625	. . G G G G    
626	. . G G G G    
627	. . G G G G    
628	. . A A A A    
629	. . G G G G    
630	. . T T T T    
631	. . G G G G    
632	. . A A A A    
633	. . T T T T    
634	. . T T T T    
635	. . C C C C    
636	. . A A A A    
637	. . T T T T    
638	. . T T T T    
639	. . G G G G    
640	. . A A A A    
641	. . C C C C    
642	. . C C C C    
643	. . T T T T    
644	. . G G G G    
645	. . G G G G    
646	. . G G G G    
647	. . A A A A    
648	. . C C C C    
649	. . T T T T    
650	. . G G G G    
651	. . G G G G    
652	. . G G G G    
653	. . A A A A    
654	. . G G G G    
655	. . T T T T    
656	. . T T T T    
657	. . G G G G    
658	. . A A A A    
659	. . T T T T    
660	. . . T T T    
661	. . . C C C    
662	. . . A A A    
663	. . . T T T    
664	. . . T T T    
665	. . . G G G    
666	. . . A A A    
667	. . . C C C    
668	. . . T T T    
669	. . . T T T    
670	. . . A A A    
671	. . . G G G    
672	. . . G G G    
673	. . . A A A    
674	. . . C C C    
675	. . . T T T    
676	. . . G G G    
677	. . . G G G    
678	. . . G G G    
679	. . . A A A    
680	. . . G G G    
681	. . . T T T    
682	. . . T T T    
683	. . . G G G    
684	. . . A A A    
685	. . . T T T    
686	. . . T T T    
687	. . . C C C    
688	. . . A A A    
689	. . . T T T    
690	. . . T T T    
691	. . . G G G    
692	. . . A A A    
693	. . . C C C    
694	. . . C C C    
695	. . . T T T    
696	. . . G G G    
697	. . . G G G    
698	. . . G G G    
699	. . . A A A    
700	. . . C C C    
701	. . . T T T    
702	. . . G G G    
703	. . . G G G    
704	. . . G G G    
705	. . . A A A    
706	. . . G G G    
707	. . . T T T    
708	. . . T T T    
709	. . . G G G    
710	. . . A A A    
711	. . . T T T    
712	. . . T T T    
713	. . . C C C    
714	. . . A C C    
715	. . . . C C    
716	. . . . C C    
717	. . . . . A    
718	. . . . . A    
719	. . . . . G    
720	. . . . . G
```
